# Address

**Published:** 1895-08

**Authors:** S. Stewart


### ADDRESS.[1]

BY S. STEWART, M.D., D.V.M.

THIS is an age of progress, in which it is our privilege to inhabit the earth, and we are in duty bound by the spirit of our times to take a part in the progress and uplift of humanity. As we are privileged to enjoy the fruits of the present high plane of civilization which has been wrought out of so much ignorance, venality, pain, and death, the obligation rests upon us to do all we can to lessen the ignorance and reduce the venality which still entail pain and death. We, as veterinarians, have a special opportunity through the rapidly-enlarging field of sanitary science to render valuable service to mankind, and will prove ourselves very derelict and unworthy if we fail to improve this golden opportunity. The veterinary profession has already rendered, and is now rendering, great service to the medical profession in demonstrating the relation of diseases of animals to diseases of mankind, and this service is duly recognized by the teachers of human medicine, but the fulness of this great

[1] President's Address, read before the Missouri Valley Medical Association, October, 1894.

work is not completed. It is not enough to show that the tubercle bacilli of animals and mankind are the same; that the actinomyces which grow in the jaw of an ox are identical with the actino which grows in the jaw or lungs of a man; that glanders or anthrax germs may be communicated from animals to man and produce death. The veterinarian should see to it that his community is made aware of these facts, and when it is better informed it will establish regulations to prevent ignorance and knavery from selling diseased and impure meat and milk for public consumption.

Did the public fully comprehend that to have pure and wholesome milk the animal producing it must be free from disease, must have pure food to eat, pure water to drink, and a clean inclosure to stay in; that to have wholesome meat food-animals must be environed by the same condition—the tuberculous cow would be banished, spoiled or diseased provender would be unsalable, and the product of fattening-pens supplied with foul water would fail to find a market. All these things are to be brought about by sanitary education, and veterinarians are the rightful educators. The people will look to them for knowledge of these things and are entitled to expect and receive it from them.

Now this is not mere fancy and figure of speech, but the simple truth. You may query how is all this to be accomplished, by what means can it be brought about, what can I do to help on this noble and glorious work. Let us see. The great majority of our people read the public press, and, were the many phases of this broad subject brought to their notice through the columns of the local papers in your town and mine, a gradually enlarging concept of sanitation as applied to these would manifest itself. First individuals, then the whole community, would require that wholesome meat and milk be supplied, would refuse to accept any other, and would secure competent veterinary officers to use their knowledge for the protection of the public.

Yes, you say, that is all very nice, but how can I use the public press to bring about these results and not sully my character as a scientific man, not violate the ethics of veterinary gentlemen, and not assume the practices of the charlatan? If I am rightly informed, it is the special reference to the individual veterinarian or special reference to a given remedy or operation used by him, perhaps by him only, which appeared in the columns of the local press, that renders the publication dishonorable, that violates the ethics of the profession, that reflects the charlatan. These special references can be put one side ; the writer's name can be omitted when published at home, if by publishing the name a slight may be cast on a neighboring veterinarian. Many articles well calculated to arouse and educate are to be found in papers, journals, and books published elsewhere, which, if brought to the attention of the local newspaper man, would readily find place in the columns of his paper, and in this way, though you do not want to write for publication yourself, you can provide a large and useful variety of articles bearing on this subject for the people of your locality to read.

It is proper and right to talk sanitation to your patrons, to your neighbors, to the physician of your locality, to the members of the local board of health, to an association of stock-breeders, to a dairymen's association, and to public gatherings for the consideration of sanitary subjects, so long as the *ego* is kept in the background.

You may help on this good cause in yet another way ; you can examine your patron's farm, stable, stock, provender, and water-supply, and explain to him these sanitary truths, when he will at once become a disciple and aid you in this good work. You can render yourself competent to examine the local dairies and take charge of the inspection of local or public abattoirs and render service to the community in a larger way. When the community has acquired the proper information it will seek your services as a sanitarian. It is your privilege and opportunity to bring both these results to pass.

It is surely very gratifying to all that so many veterinarians of this section should deem it good to meet at this appointed time and place. It means very much for professional uplift and professional growth in this community. It is indeed a pleasure to welcome you to this assemblage, and it is our sincere hope that the interchange of greetings may establish an acquaintance of each with the other ; that the exchange of thoughts on topics in which we are all interested may develop into mutual good-fellowship, into professional fraternity in the full meaning of the word. It is fitting that it should be so. Why should it be otherwise? The history of our profession in American cities is largely one of professional discredit, one would almost be justified in saying dishonor. Bitter jealousy, contention, and strife are the prevailing conditions. Until within the past year in no cities, excepting Philadelphia and Boston, have the veterinarians been able to maintain an association, and why? Let me tell you. The profession has put a wrong definition on the word success. It has been considered to mean dollars, and dollars only; everything else has been sacrificed to Mammon. Honor, integrity, friendship, brotherly love, and professional growth have had no relation to the idea of success. What think you of a veterinarian who has secured many dollars, but no professional friends? What think you of the man who has traded on his professional credentials and made no scientific growth? What think you of the veterinary surgeon who has no words of good cheer for a neighboring V.S., nor a word of charity for his mistakes? Let us define anew that word success. Let us make it include the esteem of our professional brethren, make it include scientific attainment, make it include doing unto each other as we would like to be done by. Be it remembered that it is becoming in any man to forgive the mistakes and ethical transgressions of another, if he himself would be forgiven. A lack of ethical knowledge and integrity has much to do with professional discord. Let us cultivate this phase of our professional life and bind together the members of this Association with the bands of fraternity and good-fellowship, then will we make scientific growth possible and certain.

The Committee on Constitution and By-laws will offer for your consideration and adoption a code of rules to serve, as are outlined, for the maintenance of right relations with each other; but let us learn to be more than is prescribed therein. Let us learn to be rejoiced with a brother's success and grieved by his failure; let us learn to lend a helping hand in his time of trouble and give an encouraging word for better endeavor; let us learn to counsel with one another, and resist the temptation to steal the other's client, and injure his reputation. Then may we claim true progress in our profession and be assured of true success.

---

Drs. Rectenwald and Waugh, of Pittsburg, recently entered suit against two travelling veterinary dentists, practising in Allegheny County contrary to the laws of Pennsylvania. Before the hearing the violators took flight for other fields. A few more efforts of this kind will teach a wholesome respect for the law.

